# Resist antibiotic use before resistance spreads

**DOI:** 10.1186/1865-1380-7-S1-P7

**Published:** 2014-07-25

**Authors:** Kiruthigha Kaliyamoorthy, Nithya Ganesan, Aditya Maddali, Tausif Thangalvadi, Parivalavan Rajavelu

**Affiliations:** 1 Sundaram Medical Foundation, Dr. Rangarajan Memorial hospital, Shanthi Colony, Anna Nagar, Chennai, India

## Objective

Urinary tract infection (UTI) is one of the common complaints with which patients present to the emergency department. There are very few studies from our part of the country for UTI. The most commonly used antibiotics are fluoroquinolones, nitrofurantoin and aminoglycosides. The objective of this study was to evaluate the characteristics of bacteria involved in UTI and the prevalent antibiotic resistance patterns.

## Methods

A retrospective study was performed to investigate the possible association between prescribed antibiotics and resistance of organisms involved in UTI. Patients above the age of 18 who presented to the ED with diagnosis of suspected UTI were included from November 2012 to January 2013. Patients below the age of 18, patients with genitourinary abnormalities including previous genitourinary surgery, patients in septic shock or those with a history of sexual abuse were excluded.

## Results

Out of 207 patients, 34 (16%) had positive urine routine, 16 (7%) culture positive and 52 (25%) were positive for both. Commonly encountered organisms were *E.Coli* (77%), Pseudomonas (4%), Klebsiella (5%) and Enterococcus (5%). *E.coli* was found resistant to common antibiotics like Nitrofurantoin (69%), Norfloxacin (32%), Amoxycillin (52%), Gentamycin (52%) and Cefoperazone sulbactam (35%). Klebsiella had resistance to Norfloxacin (2%), Ciprofloxacin (4%), Gentamycin (4%) and Cotrimoxazole (4%). Pseudomonas was resistant to Ciprofloxacin (4%), Ofloxacin (2%), Cefoperazone sulbactam (4%) and Gentamycin (4%). Resistance to Nirtofurantoin (2%), Ofloxacin (1.4%), Ciprofloxacin (1.4%) and Amoxicillin (4%) was noted in Enterococcus.

## Conclusion

The most common organism noted in UTIs is *E.coli.* High resistance is noted to commonly prescribed antibiotics probably secondary to antibiotic abuse in the community. Knowledge of resistance patterns will help us use antibiotics judiciously to combat this concerning issue. Antibiotic stewardship is the need for the day.

